# Air pollution increases the risk of SSNHL: A nested case-control study using meteorological data and national sample cohort data

**DOI:** 10.1038/s41598-019-44618-0

**Published:** 2019-06-04

**Authors:** Hyo Geun Choi, Chanyang Min, So Young Kim

**Affiliations:** 1 Department of Otorhinolaryngology-Head & Neck Surgery, Hallym University College of Medicine, Anyang, Korea; 2 Hallym Data Science Laboratory, Hallym University College of Medicine, Anyang, Korea; 3 Graduate School of Public Health, Seoul National University, Seoul, Korea; 4 Department of Otorhinolaryngology-Head & Neck Surgery, CHA Bundang Medical Center, CHA University, Seongnam, Korea

**Keywords:** Comorbidities, Risk factors

## Abstract

This study aimed to evaluate the impact of weather conditions and air pollution on the onset of sudden sensorineural hearing loss (SSNHL). The Korean Health Insurance Review and Assessment Service - National Sample Cohort (HIRA-NSC) from 2002 through 2013 was used. A total of 5,200 participants with SSNHL were matched 1:4 for age, sex, income, region of residence, hypertension, diabetes, and dyslipidemia with 20,800 control participants. Meteorological data included daily mean temperature (°C), daily highest temperature (°C), daily lowest temperature (°C), daily temperature difference (°C), relative humidity (%), ambient atmospheric pressure (hPa), pressure, SO_2_ (ppm), NO_2_ (ppm), O_3_ (ppm), CO (ppm), and PM_10_ (μg/m^3^) of a mean of 60 days, 30 days, 14 days, 7 days, and 3 days before SSNHL were analyzed. Hourly measurements were taken from 94 places to assess the temperature, humidity, and atmospheric pressure and from 273 places to determine SO_2_, NO_2_, O_3_, CO, and PM_10_. Crude and adjusted odds ratios (ORs) and 95% confidence intervals (CIs) of meteorological data for SSNHL were analyzed using unconditional logistic regression analyses. Subgroup analyses were conducted by age and sex. The mean NO_2_ and O_3_ concentrations 14 days before the index date were different in the SSNHL group compared to those in the control group (P < 0.001 for NO_2_ and P = 0.021 for O_3_). The adjusted 14-day OR for NO_2_ (0.1 ppm) exposure was 3.12 in the SSNHL group compared to that in the control group (95% CI = 2.16–4.49, P < 0.001). The increased odds of NO_2_ exposure for 14 days in the SSNHL group persisted in the age group older than 30 years for both sexes. Other meteorological conditions did not show differences between the SSNHL and control groups. SSNHL was associated with high concentrations of NO_2._

## Introduction

Industrialization has contributed to increasing health and economic burdens from air pollution^1^. Air pollutants, including particulate matter (PM), nitrogen oxide (NO_2_), and ozone (O_3_), impact extrapulmonary and pulmonary systems^2^. Cardiovascular disorders, such as acute myocardial infarction and stroke, are linked to increased levels of air pollutants^3–6^. Previous studies found that elevated concentrations of PM_10_ or NO_2_ were associated with acute myocardial infarction and ischemic stroke^3,6^. Many cohort studies have demonstrated that the air pollutants SO_2_, NO_2_, and PM_10_ are associated with elevated cardiovascular mortality^5^. In addition, the air pollutant NO_2_ and the oxidative potential of PM_2.5_ contribute to an increased risk of diabetes^7^. Because air pollutants are exposed as compounds under consistently changing weather conditions, multiple factors need to be considered to investigate their health effects. When exploring the impact of air pollutants on specific diseases, considering conditions such as temperature is crucial because it determines the concentration of air pollutants. For instance, the concentration of O_3_ peaks when the temperature is highest^4^. Therefore, this study included constant evaluations of both weather conditions and multiple air pollutant exposures to identify unbiased effects.

Sudden sensorineural hearing loss (SSNHL) is defined as sensorineural hearing loss with sudden onset^8^. Approximately 35–68% of SSNHL patients had permanent hearing loss in spite of steroid and other treatments^9^. Approximately 27 per 100,000 persons suffer from SSNHL in the United States each year. In Korea, the incidence of SSNHL was estimated to be approximately 17.76 per 100,000 persons per year^10^. The cause of SSNHL is elusive and multifactorial. A viral etiology has been suggested with evidence obtained from clinical cases and from temporal bone pathological findings^11^.

Because viral infection can be influenced by meteorological conditions, a few previous studies proposed an association between SSNHL and meteorological conditions with conflicting results^12,13^. A retrospective study of hospital patients reported that, of the different meteorological conditions, the onset of SSNHI was associated only with strong wind speeds for 7 days^12^. Another retrospective study described no significant relationship between the onset of SSNHL and any meteorological conditions, including temperature and atmospheric pressure^13^. In addition, several recent studies have identified cardiovascular causes of SSNHL^14,15^. Because cardiovascular diseases are influenced by air pollution, air pollution might have an impact on SSNHL^16^. Furthermore, a number of recent studies demonstrated an association between hearing loss and air pollutants from cigarette smoking^17,18^. Current smokers had 1.15 times higher odds of developing hearing loss than nonsmokers (95% confidence intervals [95% CI = 1.09–1.21])^18^. However, few studies have investigated the impact of air pollution on SSNHL. When the PubMed and EMBASE databases were searched for studies using the keyword phrase ‘(sudden sensorineural hearing loss) AND (pollution)’, no article was retrieved until September 2018.

The present study hypothesized that meteorological conditions (including air pollution) can influence the onset of SSNHL. To confirm this hypothesis, differences in meteorological conditions were analyzed between the SSNHL and the control group.

## Results

Age, sex, income level, region of residence, and past medical histories of hypertension, diabetes, and dyslipidemia were precisely matched between the SSNHL and control groups. We described the mean of meteorological and air pollution measurements for 14 days before the index date. Only NO_2_ and O_3_ were significantly different (Table 1, P < 0.001 for NO_2_ and P = 0.021 for O_3_).

**Table 1:** General Characteristics of Participants.

Characteristics	Total participants		
	Sudden sensory neural hearing loss	Control group	P-value
Age (years old, n, %)			1.000
5–9	27 (0.5)	108 (0.5)	
10–14	65 (1.3)	230 (1.3)	
15–19	138 (2.7)	552 (2.7)	
20–24	149 (2.9)	596 (2.9)	
25–29	254 (4.9)	1,016 (4.9)	
30–34	304 (5.8)	1,216 (5.8)	
35–39	413 (7.9)	1,652 (7.9)	
40–44	480 (9.2)	1,920 (9.2)	
45–49	529 (10.2)	2,116 (10.2)	
50–54	642 (12.3)	2,568 (12.3)	
55–59	599 (11.5)	2,396 (11.5)	
30–64	511 (9.8)	2,044 (9.8)	
65–69	461 (8.9)	1,844 (8.9)	
70–74	342 (6.6)	1,368 (6.6)	
75–79	187 (3.6)	748 (3.6)	
80–84	69 (1.3)	276 (1.3)	
85+	30 (0.6)	120 (0.6)	
Sex (n, %)			1.000
Male	2,304 (44.3)	9,216 (44.3)	
Female	2,896 (55.7)	11,584 (55.7)	
Income (n, %)			1.000
1 (lowest)	88 (1.7)	352 (1.7)	
2	346 (6.7)	1,384 (6.7)	
3	310 (6.0)	1,240 (6.0)	
4	337 (6.5)	1,348 (6.5)	
5	325 (6.3)	1,300 (6.3)	
6	437 (8.4)	1,748 (8.4)	
7	459 (8.8)	1,836 (8.8)	
8	542 (10.4)	2,168 (10.4)	
9	620 (11.9)	2,480 (11.9)	
10	835 (16.1)	3,340 (16.1)	
11 (highest)	901 (17.3)	3,304 (17.3)	
Region of residence (n, %)	2,430 (46.7)	9,720 (46.7)	1.000
Hypertension (n, %)	1,930 (37.1)	7,720 (37.1)	1.000
Diabetes (n, %)	1,139 (21.9)	4,556 (21.9)	1.000
Dyslipidemia (n, %)	1,636 (31.5)	6,544 (31.5)	1.000
Daily mean temperature for 14 days (°C, mean, SD)	13.0 (9.7)	13.1 (9.6)	0.821
Daily highest temperature for 14 days (°C, mean, SD)	18.2 (9.5)	18.2 (9.4)	0.946
Daily lowest temperature for 14 days (°C, mean, SD)	8.6 (10.1)	8.7 (10.1)	0.804
Daily temperature difference for 14 days (°C, mean, SD)	9.6 (2.3)	9.6 (2.3)	0.417
Relative humidity for 14 days (%, mean, SD)	65.6 (10.6)	65.8 (10.6)	0.467
Ambient atmospheric pressure for 14 days (hPa, mean, SD)	1006.3 (7.5)	1006.1 (7.6)	0.078
SO_2_ for 14 days (ppb, mean, SD)	5.5 (1.9)	5.5 (2.0)	0.851
NO_2_ for 14 days (ppb, mean, SD)	24.9 (8.8)	24.1 (8.6)	<0.001*
O_3_ for 14 days (ppb, mean, SD)	23.1 (8.7)	23.4 (8.7)	0.021*
CO for 14 days (ppm, mean, SD)	0.566 (0.181)	0.562 (0.186)	0.148
PM_10_ for 14 days (μg/m^3^, mean, SD)	52.4 (18.1)	52.1 (18.1)	0.209

SD: standard deviation.

ppb: Parts per billion.

ppm: Part per million ( = 1,000 ppb).

*Chi-square test or independent t-test, significance at P < 0.05.

The adjusted 14-day OR for NO_2_ (0.1 ppm) exposure for the SSNHL group was 3.12 (95% CI = 2.16–4.49, P < 0.001, Table 2). The daily mean temperature, daily highest temperature, daily lowest temperature, daily temperature difference, relative humidity, ambient atmospheric pressure, SO_2_, CO, and PM_10_ did not reach statistical significance (Table 3). We excluded O_3_ because it was associated with NO_2_ (Supplemental Table 1).

**Table 2:** Adjusted odds ratios (95% confidence intervals) of NO_2_ for 14 days (0.1 ppm) for sudden sensory neural hearing loss in total and subgroup analyses according to age and sex.

Participants	N (participants)	Sudden sensory neural hearing loss	
		AOR of NO_2_	P-value
Total	26,000	3.12 (2.16–4.49)	<0.001*
Age (<30 years old), men	1,520	2.05 (0.45–9.36)	0.354
Age (<30 years old), women	1,645	0.83 (0.19–3.61)	0.803
Age (30–59 years old), men	6,690	3.64 (1.76–7.50)	<0.001*
Age (30–59 years old), women	8,145	3.96 (2.07–7.56)	<0.001*
Age (≥60 years old), men	3,310	4.06 (1.41–11.61)	0.009*
Age (≥60 years old), women	4,690	2.56 (1.08–6.06)	0.032*

*Logistic regression model adjusted model for age, sex, income, region of residence, hypertension, diabetes, and dyslipidemia, significance at P < 0.05.

**Table 3:** Crude odds ratios (95% confidence intervals) of the meteorological and pollution matter for sudden sensory neural hearing loss.

Characteristics	Sudden sensory neural hearing loss	
	Crude OR (95% CI)	P-value
Daily mean temperature for 60 days (°C)	1.00 (1.00–1.00)	0.793
Daily mean temperature for 30 days (°C)	1.00 (1.00–1.00)	0.816
Daily mean temperature for 14 days (°C)	1.00 (1.00–1.00)	0.821
Daily mean temperature for 7 days (°C)	1.00 (1.00–1.00)	0.748
Daily mean temperature for 3 days (°C)	1.00 (1.00–1.00)	0.770
Daily highest temperature for 60 days (°C)	1.00 (1.00–1.00)	0.924
Daily highest temperature for 30 days (°C)	1.00 (1.00–1.00)	0.964
Daily highest temperature for 14 days (°C)	1.00 (1.00–1.00)	0.946
Daily highest temperature for 7 days (°C)	1.00 (1.00–1.00)	0.806
Daily highest temperature for 3 days (°C)	1.00 (1.00–1.00)	0.800
Daily lowest temperature for 60 days (°C)	1.00 (1.00–1.00)	0.760
Daily lowest temperature for 30 days (°C)	1.00 (1.00–1.00)	0.771
Daily lowest temperature for 14 days (°C)	1.00 (1.00–1.00)	0.804
Daily lowest temperature for 7 days (°C)	1.00 (1.00–1.00)	0.765
Daily lowest temperature for 3 days (°C)	1.00 (1.00–1.00)	0.788
Daily temperature difference for 60 days (°C)	1.01 (0.99–1.02)	0.284
Daily temperature difference for 30 days (°C)	1.01 (0.99–1.02)	0.242
Daily temperature difference for 14 days (°C)	1.01 (0.99–1.02)	0.417
Daily temperature difference for 7 days (°C)	1.00 (0.99–1.01)	0.783
Daily temperature difference for 3 days (°C)	1.00 (0.99–1.01)	0.915
Relative humidity for 60 days (%)	1.00 (1.00–1.00)	0.436
Relative humidity for 30 days (%)	1.00 (1.00–1.00)	0.385
Relative humidity for 14 days (%)	1.00 (1.00–1.00)	0.467
Relative humidity for 7 days (%)	1.00 (1.00–1.00)	0.885
Relative humidity for 3 days (%)	1.00 (1.00–1.00)	0.950
Ambient atmospheric pressure for 60 days (hPa)	1.00 (1.00–1.00)	0.067
Ambient atmospheric pressure for 30 days (hPa)	1.00 (1.00–1.00)	0.074
Ambient atmospheric pressure for 14 days (hPa)	1.00 (1.00–1.01)	0.078
Ambient atmospheric pressure for 7 days (hPa)	1.00 (1.00–1.01)	0.079
Ambient atmospheric pressure for 3 days (hPa)	1.00 (1.00–1.01)	0.090
SO_2_ for 60 days (0.1 ppm)	0.99 (0.10–5.11)	0.989
SO_2_ for 30 days (0.1 ppm)	1.16 (0.24–5.63)	0.851
SO_2_ for 14 days (0.1 ppm)	1.16 (0.25–5.31)	0.853
SO_2_ for 7 days (0.1 ppm)	1.15 (0.27–4.94)	0.851
SO_2_ for 3 days (0.1 ppm)	1.01 (0.27–3.77)	0.992
NO_2_ for 60 days (0.1 ppm)	2.84 (1.96–4.11)	<0.001*
NO_2_ for 30 days (0.1 ppm)	2.81 (1.97–4.02)	<0.001*
NO_2_ for 14 days (0.1 ppm)	2.77 (1.96–3.91)	<0.001*
NO_2_ for 7 days (0.1 ppm)	2.46 (1.77–3.41)	<0.001*
NO_2_ for 3 days (0.1 ppm)	2.16 (1.61–2.89)	<0.001*
O_3_ for 60 days (0.1 ppm)	0.63 (0.43–0.93)	0.020*
O_3_ for 30 days (0.1 ppm)	0.64 (0.45–0.93)	0.018*
O_3_ for 14 days (0.1 ppm)	0.66 (0.47–0.94)	0.021*
O_3_ for 7 days (0.1 ppm)	0.70 (0.50–0.98)	0.037*
O_3_ for 3 days (0.1 ppm)	0.75 (0.55–1.02)	0.070
CO for 60 days (1 ppm)	1.11 (0.93–1.33)	0.243
CO for 30 days (1 ppm)	1.11 (0.94–1.32)	0.226
CO for 14 days (1 ppm)	1.13 (0.96–1.33)	0.148
CO for 7 days (1 ppm)	1.12 (0.96–1.31)	0.140
CO for 3 days (1 ppm)	1.13 (0.98–1.29)	0.096
PM_10_ for 60 days (10 μg/m^3^)	1.02 (0.99–1.04)	0.164
PM_10_ for 30 days (10 μg/m^3^)	1.00 (1.00–1.00)	0.162
PM_10_ for 14 days (10 μg/m^3^)	1.00 (1.00–1.00)	0.209
PM_10_ for 7 days (10 μg/m^3^)	1.00 (1.00–1.00)	0.291
PM_10_ for 3 days (10 μg/m^3^)	1.00 (1.00–1.00)	0.332

*Logistic regression model, significance at P < 0.05.

We analyzed the odds ratios of meteorological data for sudden sensory neural hearing loss using simple logistic regression analysis. In these results, only NO_2_ and O_3_ showed statistical significance (P < 0.05). Therefore, we chose these NO_2_ and O_3_ as the independent variables.

In subgroup analyses, NO_2_ (0.1 ppm) measured over 14 days increased the risk of SSNHL in 30–59-year-old men (AOR = 3.64, 95% CI = 1.76–7.50, P < 0.001) and women (AOR = 3.96, 95% CI = 2.07–7.56, P < 0.001) and in men 60 years or older (AOR = 4.06, 95% CI = 1.41–11.61, P = 0.009) as well as women (AOR = 2.56, 95% CI = 1.08–6.06, P = 0.032) (Table 2). However, these associations did not reach statistical significance among participants younger than 30 years old for both men and women.

## Discussion

In the present study, SSNHL patients demonstrated a higher odds of NO_2_ exposure than the controls (adjusted OR = 3.12, 95% CI = 2.16–4.49, P < 0.001). Other meteorological factors, including temperature, humidity, and atmospheric pressure, as well as air pollutants of SO_2_, CO, and PM_10_, did not show a significant difference between the SSNHL and control groups.

Systemic inflammation and oxidative stress induced by NO_2_ could increase the risk of SSNHL. Inflammation and oxidative stress are also known to be related to SSNHL^19^. NO_2_ has been shown to evoke an inflammatory response and to increase susceptibility to infection even in healthy subjects^2^. The adverse health effects of NO_2_ were not limited to the duration and amount of exposure, as concluded in a previous review^20^. A short-term exposure is defined as being exposed to 50 µg NO_2_/m^3^ in less than 24 hours, which is associated with an increased rate of hospital admissions and mortality^20^. In addition, a low concentration below 40 µg NO_2_/m^3^ has also been correlated with adverse health outcomes (respiratory diseases, hospital admissions, mortality, and otitis media)^20^.

NO_2_ influences intracochlear nitric oxide (NO) concentration, which leads to an alteration in intracochlear neurotransmission and neuromodulation. NO plays a crucial role as a signaling molecule in gap junctions, blood vessels, and the synaptic region of the cochlea^21^. Thus, elevated NO concentrations can result in hearing impairment^21^. Similarly, the modulation of the intracochlear NO concentration might influence the risk of SSNHL.

In this study, the cumulative influences of NO_2_ on SSNHL can be postulated from the lag effects of the 14-day NO_2_ concentrations. Although the concentration of NO_2_ at 60, 30, 14, 7, and 3 days before the onset of SSNHL was related to SSNHL, the concentrations of NO_2_ 14 days before the onset of SSNHL were the smallest values based on the Akaike and Baysian information criteria. A previous study reported that the long-term exposure to low-concentration NO_2_ was related to adverse health outcomes (respiratory diseases, hospital admissions, mortality, and otitis media)^20^. Moreover, the latency of viral infections could influence the lag effects of NO_2_ on SSNHL. A population cohort study reported that the lag effects of NO_2_ were a risk factor for acute upper respiratory infections^22^. The cumulative 6-day NO_2_ concentration increased the risk of acute upper respiratory infection (relative risk = 1.25, 95% CI = 1.21–1.29)^22^. Because viral infection is one of the risk factors for SSNHL^23^, the lag effects of NO_2_ on viral infections might affect the lag effects of NO_2_ on SSNHL observed in this study.

The effect of NO_2_ on SSNHL was independent of other air pollutants in this study. However, the effects of NO_2_ on SSNHL could represent the composite effects of air pollutants on SSNHL because NO_2_ is an indicator of air pollution from traffic in urban areas. Nonetheless, NO_2_ has been proposed to be an independent contributor to increased cardiovascular and respiratory mortality^24,25^. A meta-analysis reported that NO_2_ increased cardiovascular mortality by 1.13-fold (95% CI = 1.09–1.18) and respiratory mortality by 1.20-fold (95% CI = 1.09–1.31), and the results were consistent after considering the effect of PM^24^. Moreover, another study demonstrated that the effects of NO_2_ on acute myocardial infarction were higher than the effects of PM_10_ or O_3_^4^. However, other air pollutants (e.g., O_3_ and PM) were not associated with SSNHL in the present study. Although O_3_ was related to SSNHL, collinearity with NO_2_ prevented efforts to elucidate the effect of O_3_ on SSNHL. The health effects of O_3_ have been controversial in prior studies. A previous study suggested that O_3_ induced inflammation and increased the risk of lung diseases^26^. However, O_3_ also exhibited protective effects against viral infections through virucidal activity^27^. PM did not show an association with SSNHL in this study. Because the composition of PM can be different depending on the districts, the impact of PM on SSNHL might be mixed and attenuated in this study. A previous study reported that the oxidative potential of PM but not the PM itself was associated with diabetes^7^. The effects of PM on mortality outcomes (all-cause, cardiovascular, and respiratory causes) were mitigated after considering NO_2_^25^. The components of PM might have a greater influence on health than the concentration of PM.

The high odds of NO_2_ exposure in the SSNHL group were consistent in the subgroup analysis based on age and sex. Only in the group of men and women <30 years old was no association found between SSNHL and NO_2_. This might be due to the relatively small number of SSNHL participants in these young populations. A small sample size or different regional locations of the study groups and possible confounders that were not considered could all explain the different findings in previous studies. In addition, the effects of air pollutants on health problems might be more pronounced in old populations than in young populations. Prior studies have reported a greater influence of NO_2_ on acute myocardial infarction in old populations^4^. The reduced metabolism and diminished secretion abilities in older populations might increase their susceptibility to the adverse effects of air pollutants.

The weather conditions of temperature, humidity, and atmospheric pressure were not related to SSNHL in this study. Associations between SSNHL and weather conditions have been controversial. Some previous studies suggested an association between SSNHL and weather conditions^12,28^. A hospital retrospective study demonstrated that the maximum wind speed was faster within 5 days of onset of SSNHL compared to the days when SSNHL did not occur^12^. Another study reported that low atmospheric pressure was related to the onset of SSNHL^28^. However, both studies were conducted with a small number of study participants in one hospital. On the other hand, similar to the present results, there have been a few articles reporting no association between SSNHL and weather conditions^13,29^. A population cohort study in Taiwan found no evidence of an association between the onset of SSNHL and meteorological conditions of temperature, humidity, and atmospheric pressure^29^. Although temperature and humidity were related to the incidence of SSNHL before adjusting for seasonality and months, these meteorological conditions were not associated with the incidence of SSNHL after the adjustment^29^.

This study is the first to assess the association between air pollution and SSNHL. The nationwide, representative cohort population used in this study strengthens the reliability of the present results. In Korea, all the medical records of citizens are legally registered and managed by NHIS. The national health insurance system is operated based on the NHIS data. Thus, no missing participants were anticipated in the NHIS data. NHIS-NSC data were extracted by statisticians, and the representativeness of the data was verified in a previous study^30^. In addition, the equivalent control group and the adjustment of confounders also increased the reliability of this study. The demographic factors of age, sex, income, and region of residence and the past medical histories of hypertension, diabetes, and dyslipidemia were matched between the SSNHL and control groups. Because this study based on the health claim codes, the unbiased medical accessibility between study and control group was crucial. The medical accessibility was equalized by matching socioeconomic factors of income and region of residence between study and control group in this study. In addition, the medical conditions of hypertension, diabetes, and dyslipidemia were matched between study and control groups to minimize possible confounder effects. The confounding effects of these factors were not sufficiently attenuated with the adjustment in multivariable analysis in our previous study^31^. This study used the individual data by adjusting these variables, although previous studies that used Poisson analysis did not consider these individual factors. Moreover, to investigate the lag effects and to choose the most suitable models, air pollution concentrations of various durations were analyzed. The meteorological factors were measured hourly, and the daily mean values were analyzed. The accuracy of the meteorological data was guaranteed by the Korean meteorological administration. Lastly, the objective and multiple inclusion criteria for SSNHL were used in this study.

Several limitations should be considered when interpreting the present results. The degree of hearing loss varied among SSNHL participants in this study because of the lack of data regarding the severity of SSNHL in NHIS. In addition, because the diagnosis of SSNHL was based on the ICD-10 codes, it was possible to include cases of acute low frequency hearing loss, which was suggested to have different pathophysiology and prognosis^32^. Although several confounders were matched and adjusted for, the lifestyle factors of obesity, smoking, and alcohol consumption were not considered in this study. The interaction among complex mixtures of air pollutants could not be excluded, although multiple air pollutants of NO_2_, SO_2_, O_3_, and PM_10_ were considered in this study. Because PM_2.5_ has been measured since 2015 in Korea, the present study could not analyze the effect of PM_2.5_. As in other epidemiologic studies, the potential for misclassification of meteorological exposure is also possible in this study. Because meteorological exposure is estimated by residence rather than by individual patterns of activity and living circumference, the intersubject variability was feasible^33^. This study could not access information about indoor exposure to air pollutants. For instance, the indoor NO_2_ exposure from smoking, gas-fired appliances and stoves may influence the present results. Because the meteorological conditions and air pollution differ according to the region, the interpretation of this study might be limited to Korean districts. More studies in other geographical areas need to be conducted to elucidate the specific aspects of each region.

In conclusion, the mean concentration of NO_2_ before the onset of SSNHL was high in SSNHL patients. Other meteorological conditions and air pollution did not show an association with SSNHL.

## Materials and Methods

### Participant selection

The Ethics Committee of Hallym University (2017-I102) approved this study. Written informed consent was waived by the Institutional Review Board. All analyses adhered to the guidelines and regulations of the Ethics Committee of Hallym University. The Korean Health Insurance Review and Assessment Service - National Sample Cohort (HIRA-NSC), meteorological, and air pollution data are described in the supplement (Supplemental File 1).

**Figure 1:**
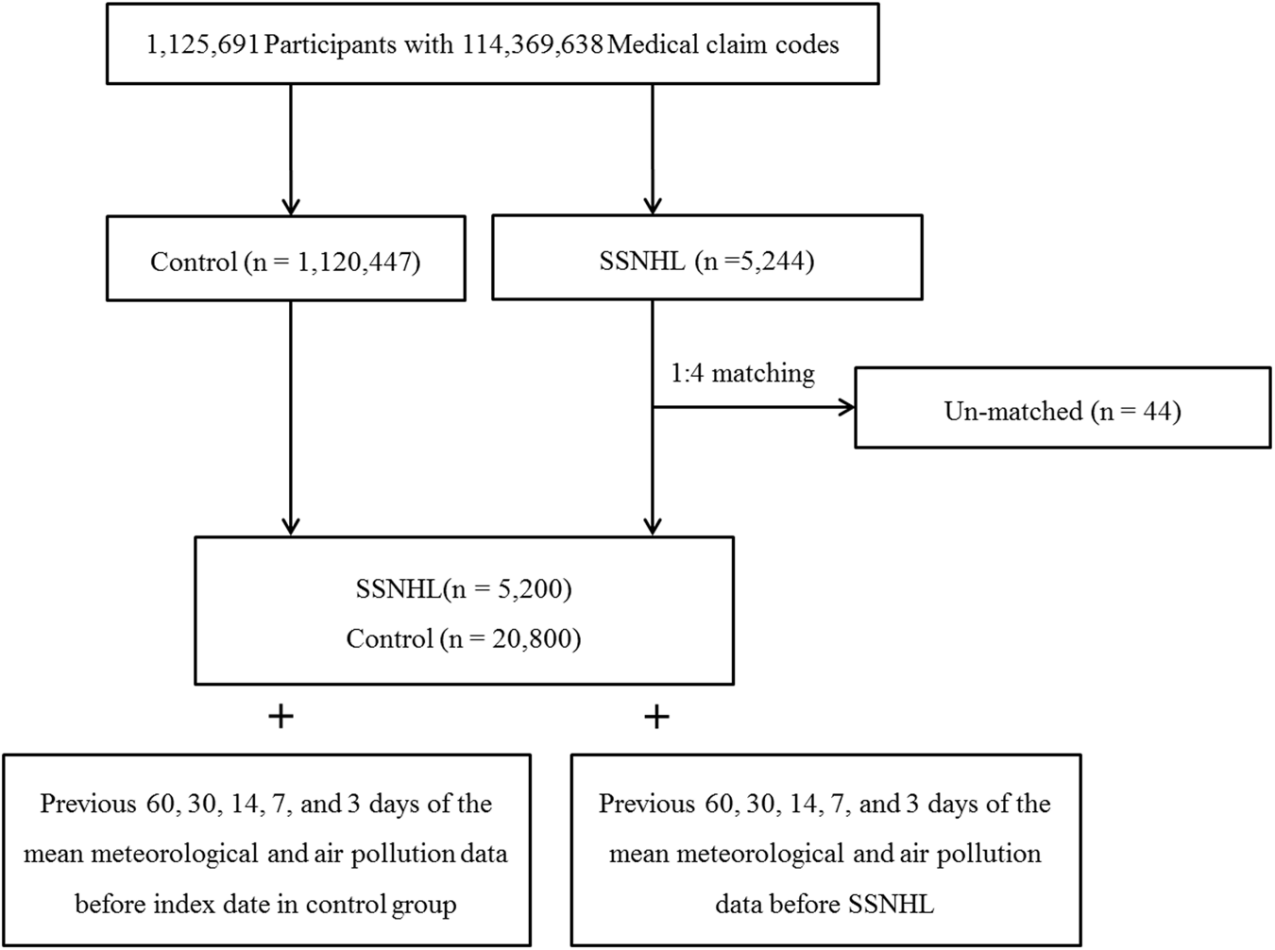
A schematic illustration of the participant selection process that was used in the present study. Of a total of 1,125,691 participants, 5,200 SSNHL participants were matched with 20,800 control participants for age, group, sex, income group, region of residence, and past medical histories. Then, SSNHL and control participants were matched with the same meteorological data before the index date.

The participants who were diagnosed with SSNHL were selected from 1,125,691 patients with 114,369,638 medical claim codes (n = 5,244). The control group included participants who were never diagnosed with SSNHL from the mother population from 2002 through 2013 (n = 1,120,447). The SSNHL and control groups were matched 1:4 for age, group, sex, income group, region of residence and for past medical histories (hypertension, diabetes, and dyslipidemia). The selection bias was minimized by selecting the control groups using a random number order process. The participants who were deceased before the index date were excluded. The index date was defined as the time when the matched SSNHL participants were included in the study. Forty-four SSNHL participants were excluded because they did not have matched control participants. Conclusively, 5,200 of SSNHL participants were matched 1:4 with 20,800 control participants (Fig. 1).

We analyzed meteorological data over a mean of 60 days, 30 days, 14 days, 7 days, and 3 days before SSNHL (index date). In the matched control group who did not experience SSNHL, we used the same matched date of SSNHL.

### Variables

#### Independent variable

Daily mean temperature (°C), daily highest temperature (°C), daily lowest temperature (°C), daily temperature difference (°C), relative humidity (%), ambient atmospheric pressure (hPa), SO_2_ (ppm), NO_2_ (ppm), O_3_ (ppm), CO (ppm), and PM_10_ (μg/m^3^) for 14 days, 10 days, 7 days, 5 days, and 3 days before the index date were defined as the independent variables (Table 3).

#### Covariate analysis

Age groups were divided into 5-year intervals: 0–4, 5–9, 10–14…, and 85+ years old. A total of 18 age groups were specified. Income groups were classified as 11 classes (class 1 [lowest income]−11 [highest income]). The region of residence was grouped into urban (Seoul, Busan, Daegu, Incheon, Gwangju, Daejeon, and Ulsan) and rural (Gyeonggi, Gangwon, Chungcheongbuk, Chungcheongnam, Jeollabuk, Jeollanam, Gyeongsangbuk, Gyeongsangnam, and Jeju) areas.

The past medical histories were collected using ICD-10 codes. Only the participants who were treated ≥2 times for hypertension (I10 and I15), diabetes (E10-E49), and dyslipidemia (E78) were included to improve the reliability of the diagnoses.

#### Dependent variable

Sudden sensory neural hearing loss (SSNHL) was selected based on ICD-10 codes (H912). We only included the participants who underwent audiometry testing (claim code: E6931-E6937, F6341-F6348) and who used steroid for treatment.

### Statistical analyses

The general characteristics between the SSNHL and control groups were compared using Chi-squared tests. The mean meteorological data from 14 days before the index date were compared using independent t-tests.

To analyze the odds ratio (OR) of meteorological data for SSNHL compared to controls, crude (simple) and adjusted (multiple) logistic regression was used and 95% confidence intervals (CIs) were calculated. The selection of independent variables and the method used to construct the final model are presented in Table 3, Supplemental Tables 1, and 2.

We calculated the single pollutant model for NO_2_, which was analyzed as the independent variable; age, sex, income, region, hypertension, diabetes, and dyslipidemia were analyzed as covariates; and SSNHL was analyzed as the dependent variable.

For the subgroup analysis, we divided participants by age and sex (young [0–29 years old], middle-aged [30–59 years old], elderly [60+ years old]; men and women). In this analysis, we used a single, combined final model.

Two-tailed analyses were performed, and significance was defined as P values less than 0.05. The SPSS version 22.0 (IBM, Armonk, NY, USA) and SAS version 9.4 (SAS Institute Inc., Cary, NC, USA) were used for the statistical analyses.

## Supplementary information

supplementary tables
