# Supplementary material for: Air pollution increases the risk of SSNHL: A nested case-control study using meteorological data and national sample cohort data
Source: Sci Rep. 2019 Jun 4;9:8270. doi: 10.1038/s41598-019-44618-0 (PMC6547844; doi:10.1038/s41598-019-44618-0)
Supplement: Supplementary file 1 — supplementary tables [file 41598_2019_44618_MOESM1_ESM.docx]

**Air pollution increases the risk of SSNHL: A nested case-control study using meteorological data and national sample cohort data**

Hyo Geun Choi, MD^1^, Chanyang Min, PhD^2,3^, So Young Kim, MD^4*^

^1^Department of Otorhinolaryngology-Head & Neck Surgery, Hallym University College of Medicine, Anyang, Korea

^2^Hallym Data Science Laboratory, Hallym University College of Medicine, Anyang, Korea

^3^Graduate School of Public Health, Seoul National University, Seoul, Korea

^4^Department of Otorhinolaryngology-Head & Neck Surgery, CHA Bundang Medical Center, CHA University, Seongnam, Korea

Running title: Nitrogen dioxide and SSNHL

*Correspondence: [sossi81@hanmail.net](mailto:sossi81@hanmail.net)

Key words: Air pollution; Nitrogen oxide; particulate matter; hearing loss

**Supplemental table 1** Correlation analysis of pollution matter

|  | NO_2_ for 14 days | O_3_ for 14 days |
| --- | --- | --- |
| NO_2_ for 14 days | 1 | -0.495* |
| O_3_ for 14 days | -0.495* | 1 |

* Significance at P < 0.01

In this analysis, the NO_2_ showed the correlation of moderate intensity (0.3 < | r | < 0.7) with O_3_.

## **Supplemental file 1 description: Study Population and Data Collection**

This national cohort study relies on data from the Korean Health Insurance Review and Assessment Service - National Sample Cohort (HIRA-NSC). The Korean National Health Insurance Service (NHIS) selects samples directly from the entire population database to prevent non-sampling errors. Approximately 2% of the samples (one million) were selected from the entire Korean population (50 million). This selected data can be classified at 1,476 levels (age [18 categories], sex [2 categories], and income level [41 categories]) using randomized stratified systematic sampling methods via proportional allocation to represent the entire population. After data selection, the appropriateness of the sample was verified by a statistician who compared the data from the entire Korean population to the sample data. The details of the methods used to perform these procedures are provided by the National Health Insurance Sharing Service. This cohort database included (i) personal information, (ii) health insurance claim codes (procedures and prescriptions), (iii) diagnostic codes using the International Classification of Disease-10 (ICD-10), (iv) death records from the Korean National Statistical Office (using the Korean Standard Classification of disease), (v) socio-economic data (residence and income), and (vi) medical examination data for each participant over a period ranging from 2002 to 2013.

Because all Korean citizens are recognized by a 13-digit resident registration number from birth to death, exact population statistics can be determined using this database. It is mandatory for all Koreans to enroll in the NHIS. All Korean hospitals and clinics use the 13-digit resident registration number to register individual patients in the medical insurance system. Therefore, the risk of overlapping medical records is minimal, even if a patient moves from one place to another. Moreover, all medical treatments in Korea can be tracked without exception using the HIRA system. In Korea, notice of death to an administrative entity is legally required before a funeral can be held. Causes of death and date are recorded by medical doctors on a death certificate.

**Meteorological Data**

Temperature (˚C), Relative humidity (%), were Spot atmospheric pressure (hPa) data were obtained from the meteorological administration. It was measured by automated synoptic observing system (ASOS) and manually in 94 places hourly. Quality was controlled following quality inspection manual (<https://data.kma.go.kr/cmmn/main.do>).

SO_2_ (ppm), NO_2_ (ppm), O_3_ (ppm), CO (ppm), and PM_10_ (μg/m3) were data were obtained by the ministry of environment. It was measured by ASOS in 273 place over the country hourly. Quality was controlled following air pollution quality control manual (<http://www.me.go.kr/home/web/index.do?menuId=10259>). We used daily mean values.

**Supplemental table 2** Akaike information criterion and Baysian information criterion of the pollution matters in crude logistic regression for sudden sensory neural hearing loss

| Pollution matters | AIC | BIC |
| --- | --- | --- |
| NO_2_ for 60 days (0.1 ppm) | 25994.762 | 26011.094 |
| NO_2_ for 30 days (0.1 ppm) | 25993.202 | 26009.534 |
| NO_2_ for 14 days (0.1 ppm) | 25991.911 | 26008.242 |
| NO_2_ for 7 days (0.1 ppm) | 25996.026 | 26012.358 |
| NO_2_ for 3 days (0.1 ppm) | 25998.568 | 26014.900 |
| O_3_ for 60 days (0.1 ppm) | 26019.490 | 26035.822 |
| O_3_ for 30 days (0.1 ppm) | 26019.312 | 26035.644 |
| O_3_ for 14 days (0.1 ppm) | 26019.574 | 26035.906 |
| O_3_ for 7 days (0.1 ppm) | 26020.558 | 26036.890 |
| O_3_ for 3 days (0.1 ppm) | 26021.632 | 26037.964 |

AIC: Akaike information criterion

BIC: Baysian information criterion

Because all of NO_2_, and O_3_ (for 60 days, 30 days, 14 days, 7 days, and 3days) showed statistical significance (S2 table), we had to choose only one of them.

In NO_2_, AIC and BIC showed the smallest value for 14 days. We chose the means of NO_2_ for 14 days before the index date.

In O_3_, AIC and BIC showed the smallest value for 30 days. However, we chose the means of O_3_ for 14 days before the index date.
